# Gallbladder tuberculosis in a dialysis patient: a case-report

**DOI:** 10.1186/s12893-020-00722-x

**Published:** 2020-04-07

**Authors:** Tewodross Getu Wolde, Kuirong Jiang, Yi Miao, Jishu Wei

**Affiliations:** 1 The First Affiliated Hospital of Nanjing Medical University, School of International Education, Nanjing Medical University, 300 Guangzhou Road, Nanjing, Jiangsu China; 2 Pancreas Center, The First Affiliated Hospital of Nanjing Medical University, 300 Guangzhou Road, Nanjing, 210029 Jiangsu China

**Keywords:** Gallbladder tuberculosis, Chronic kidney disease (CKD), End-stage renal disease (ESRD), Gallbladder cancer, Abdominal tuberculosis, Immunodeficiency, Hemodialysis

## Abstract

**Background:**

The diagnosis of gallbladder tuberculosis remains elusive even to the most experienced clinicians. Our aim is to describe our experience of this rare disease, and to raise awareness of the increasing likelihood of tuberculosis in chronic kidney disease (CKD) patient.

**Case presentation:**

We report a rare case of gallbladder tuberculosis in a chronic kidney disease patient on hemodialysis. This combination is rarely reported in literature. No signs of abdominal tuberculosis were observed besides a clinical profile consistent with CKD in our patient. The clinical signs of uremia masks those of abdominal tuberculosis and render the pre-operative diagnosis of tuberculosis more difficult.

**Conclusions:**

The clinical signs of uremia conceal those of abdominal tuberculosis. The diagnosis of tuberculosis in CKD patients hinges mainly on a high index of suspicion, perioperative findings and histological examination.

## Background

The gallbladder is an organ rarely involved in abdominal tuberculosis (TB) due to an inhibitory effect of bile on Mycobacterium bovis [1].The diagnosis of gallbladder TB remains elusive even to the most experienced clinicians. In end-stage-renal-disease (ESRD) patient with active TB, the clinical signs of uremia and TB overlap. Moreover, gallbladder TB in itself has no specific pathognomonic signs. We aim to describe our experience of this rare disease, and to raise awareness of the increasing likelihood of TB in chronic kidney disease (CKD) patients.

## Case presentation

**Fig. 1:**
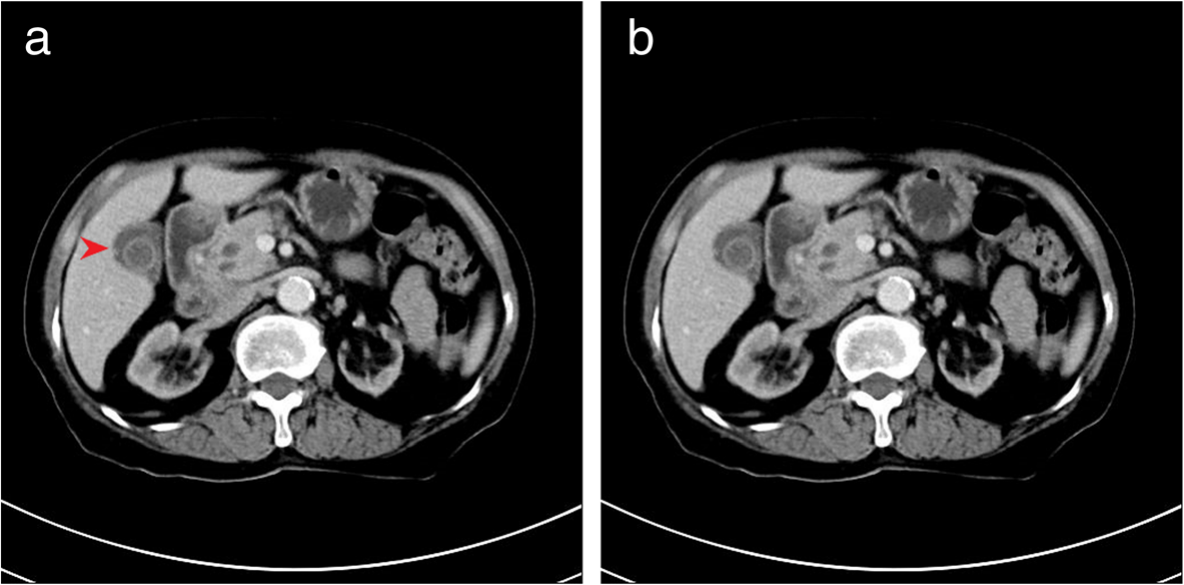
CT scan showing an increased gall bladder volume with a 1.7*1.8 cm irregular soft tissue density on the neck of the gallbladder

**Fig. 2:**
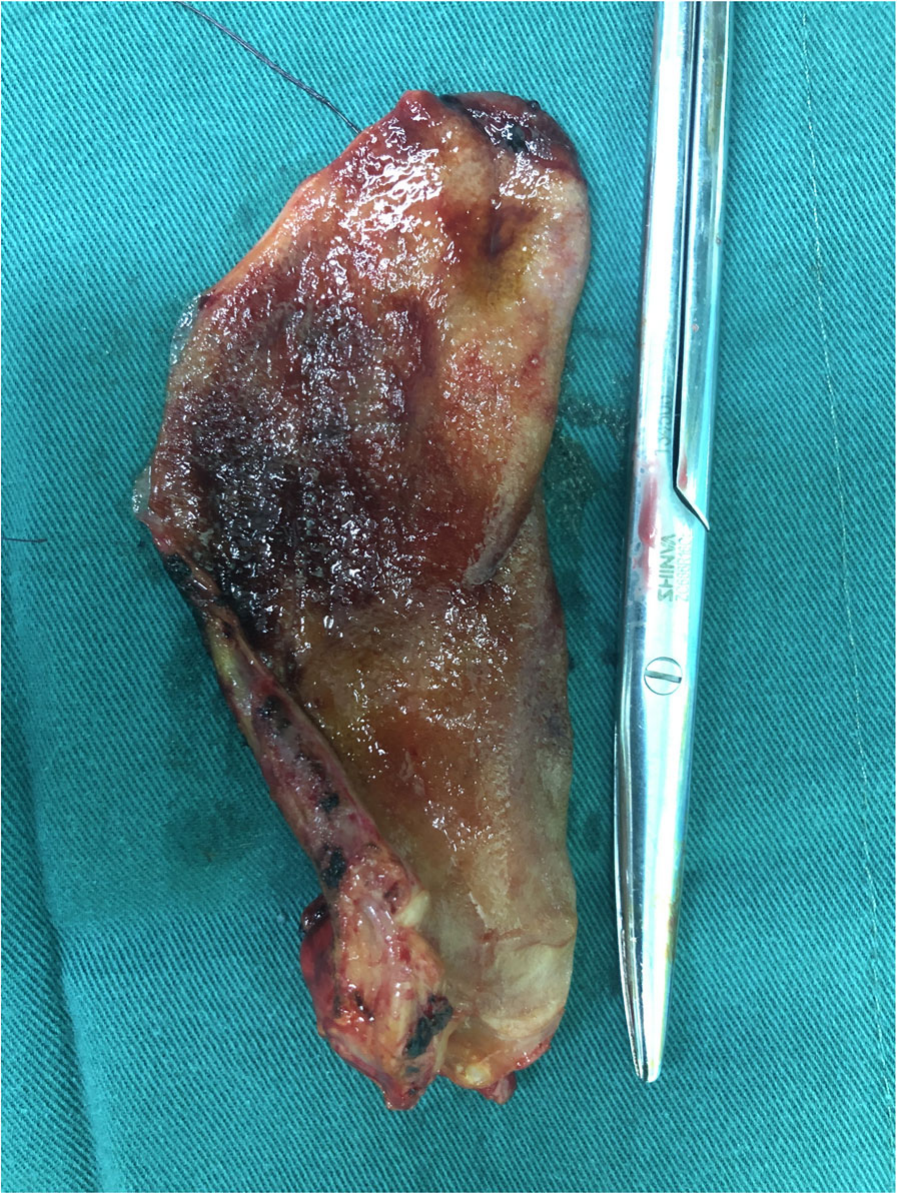
a 3*3 cm hard mass was palpated on the neck of gall bladder

**Fig. 3:**
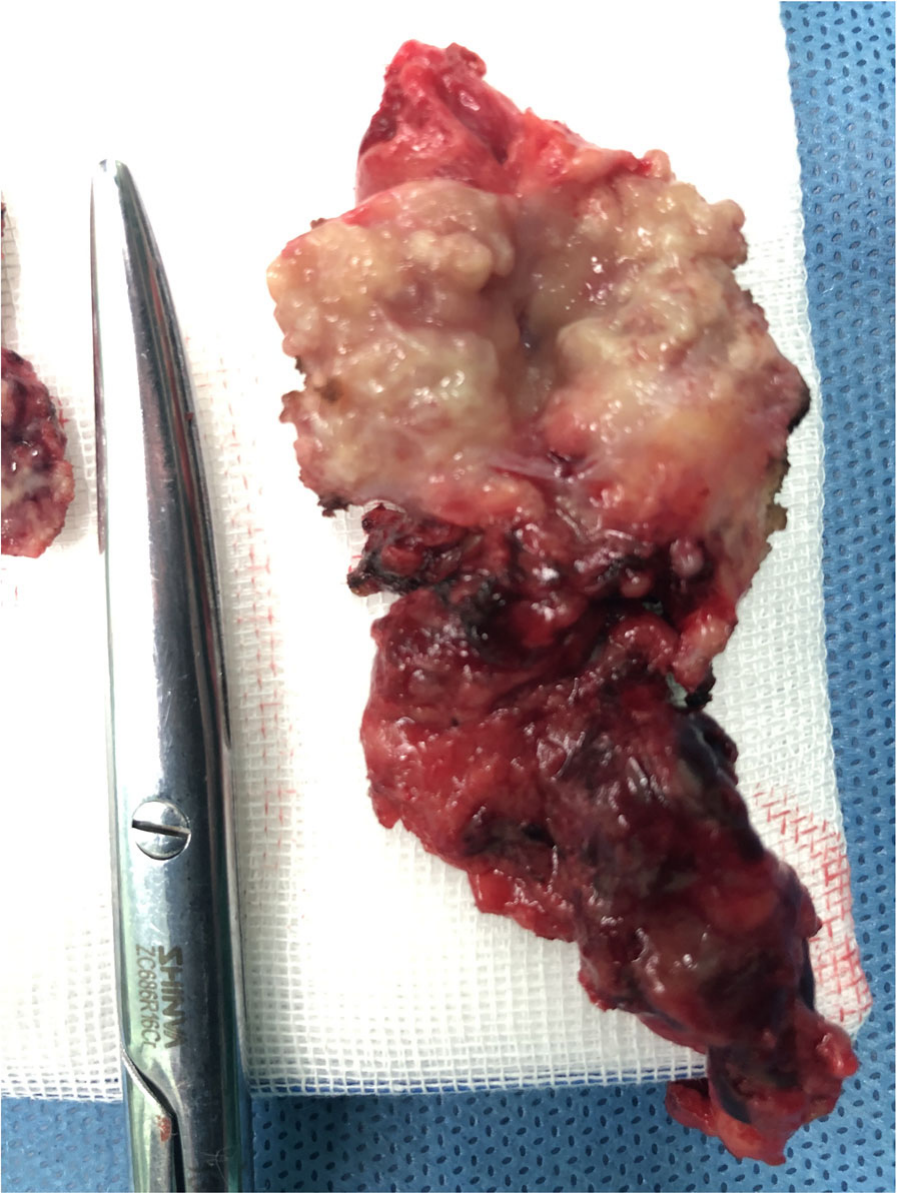
Enlarged lymph nodes resected from hepato-duodenal ligament

A 76-year old female with a 10-year history CKD presented with complaints of fatigue and anorexia. She was admitted for salvage of a malfunctioning autologous arteriovenous fistula (AVF) caused by fibro-intimal hyperplasia at the outflow vein. During hospitalization, the patient underwent a color Doppler ultrasound which revealed a thickened, 7 mm edematous gall bladder wall with accumulation of fluid in the gallbladder fossa. Computed tomography (CT) scan showed an increased gall bladder volume and a 1.7*1.8 cm irregular soft tissue density on the neck of the gallbladder (Fig. 1) with multiple small liver cysts. Few cystic lesions were noted on the head of pancreas. Bilateral renal atrophy along multiple renal calculi was also noted. The right middle lobe and lower lobe of the lung showed interstitial inflammation along with few old lesions on the right lobe. Abdominal ultrasound showed multiple hypoechoic lesions from the head of the pancreas to the hepatic hilum, indicating multiple enlarged lymph-nodes. The patient has a history of controlled hypertension. One year prior to admission, she suffered an episode of cerebral stroke without any sequelae. No family or contact history of TB was reported. The patient had generalized jaundice and scleral icterus. A complete physical examination did not reveal any other positive findings. The patient did not complain of chest tightness, nausea, emesis, dizziness or fever. Upon admission, the laboratory results were consistent with CKD and obstructive jaundice. Additionally, a high neuron specific-enolase (NSE) and Cytokeratin Fragment 2 (CYFRA) were noted, 20.03 ng/mL and 5.25 ng/mL, respectively. Tumor markers CA-19-9 and CA-125 had normal levels. The patient was scheduled for an explorative laparotomy. Upon intra-operative exploration a 3*3 cm hard mass was palpated on the neck of gallbladder with invasion into the common hepatic duct (Fig. 2). Multiple enlarged lymph nodes were observed in the hepato-duodenal ligament and behind the head of pancreas. (Fig. 3). A grayish white nodule on the liver sent for fast frozen section indicated focal necrosis and epithelioid granuloma. Histopathological examination of group 13 lymph node revealed chronic granulomatous inflammation with caseous necrosis and Langhans giant cells. While histopathological examination of the gallbladder mass showed chronic granulomatous inflammation with necrosis and multinucleated giant cells, confirming the diagnosis of gallbladder TB. However, acid-fast staining of the gallbladder mass and resected lymph nodes tested negative for bacillus. The patient underwent a radical cholecystectomy with abdominal lymphadenectomy. Following surgery, the patient was transferred to the ICU for close monitoring. The patient returned to the ward on post-operative day 1 (POD 1). A bed-side hemodialysis was regularly performed. On POD 7 the abdominal drainage tube was removed. She had an uneventful recovery and was discharged on POD 10. Subsequently, the patient was referred to a specialized center to implement an anti-tuberculosis treatment plan.

## Discussion

Gallbladder TB, owing to its rarity and non-specific manifestations, is almost indistinguishable from cancer pre-operatively. Pulmonary TB accompanied by abdominal TB is only present in 15–25% while TB of the hepato-biliary system constitutes only 1% of abdominal TB cases [2]. Various presentations of gallbladder TB have been described in many series [2–4]. However, these are neither specific nor aid in making a concrete diagnosis. The CT findings of gallbladder TB have been classified into micronodular or polypoid lesion of gallbladder wall, thickened wall-type and mass-forming type [5]. Although these imaging findings are not specific to gallbladder TB, the diagnosis of TB should be considered in patients having an irregularly thickened gallbladder wall with peripheral rim enhancement, and a gallbladder mass with or without the presence of abdominal lymphadenopathy [5]. Although the micronodular or polypoid type can imitate early gallbladder cancer on CT, it can occasionally be differentiated from carcinoma when its size is less than 1cm [5]. Preoperatively there is no definitive way to differentiate gallbladder TB from cancer, xanthogranulomatous cholecystitis and cholecystitis [1].

Paradoxically, the diagnosis of gallbladder TB is more difficult in a patient on dialysis. The performance of laboratory tests and serological TB tests are suboptimal to aid in the diagnosis of TB in a dialysis patient [6]. However, a high index of suspicion should guide the physician toward TB. CKD is in itself a state of immunosuppression. Dialysis patients are a high-risk population for TB and the incidence of TB is significantly higher in these patients [7]. Laboratory findings are generally unreliable to diagnose TB in CKD patients due to the convergence of findings of TB and uremia [6, 8]. Moreover, the clinical manifestations of uremia might hide the classical manifestations of TB. Many studies have shown the superiority of interferon-gamma-release assays (IGRA) compared to tuberculin skin test to diagnose active or latent TB (LTBI) in dialysis patients [9, 10]. The mortality rate of active TB is much higher in the dialysis population than it is the general population [11]. Due to the high prevalence of TB and the higher mortality rate of active TB in ESRD patients undergoing dialysis, it is justifiable to screen this population for LTBI to decrease the progression to active TB [12]. No specific pathognomonic signs of gallbladder TB exist, and the diagnosis of TB in CKD patients depends mainly on a high index of suspicion, perioperative findings and histological examination.

In conclusion, a thorough assessment including history, clinical manifestations, IGRA tests and chest X-rays, is essential in regions where TB is endemic, especially in patients with a history of TB and in those that are in a state of immunodeficiency.

## Data Availability

Not applicable.

## Abbreviations

CKD: Chronic Kidney Disease
ESRD: End-stage renal disease
TB: Tuberculosis
CA-19-9/125: Carbohydrate Antigen19–9/125
CYFRA: Cytokeratin Fragment
NSE: Neuron specific enzyme
POD: Post-operative day
